# Resistance to Ticks and the Path to Anti-Tick and Transmission Blocking Vaccines

**DOI:** 10.3390/vaccines9070725

**Published:** 2021-07-02

**Authors:** Jolieke G. van Oosterwijk, Stephen K. Wikel

**Affiliations:** 1 US Biologic Inc., 20 Dudley Street, Memphis, TN 38103, USA; jolieke.vanoosterwijk@usbiologic.com; 2 Department of Medical Sciences, School of Medicine, Quinnipiac University, Hamden, CT 06518, USA

**Keywords:** tick, host resistance, anti-tick vaccine, transmission blocking vaccine, immune response

## Abstract

The medical and veterinary public health importance of ticks and tick-borne pathogens is increasing due to the expansion of the geographic ranges of both ticks and pathogens, increasing tick populations, growing incidence of tick-borne diseases, emerging tick transmitted pathogens, and continued challenges of achieving effective and sustained tick control. The past decades show an increasing interest in the immune-mediated control of tick infestations and pathogen transmission through the use of vaccines. Bovine tick resistance induced by repeated infestations was reported over a century ago. This review addresses the phenomena and immunological underpinning of resistance to tick infestation by livestock and laboratory animals; the scope of tick countermeasures to host immune defenses; and the impact of genomics, functional genomics, and proteomics on dissecting complex tick–host–pathogen interactions. From early studies utilizing tick tissue extracts to salivary gland derived molecules and components of physiologically important pathways in tick gut and other tissues, an increased understanding of these relationships, over time, impacted the evolution of anti-tick vaccine antigen selection. Novel antigens continue to emerge, including increased interest in the tick microbiome. Anti-tick and transmission blocking vaccines targeting pathogen reservoirs have the potential to disrupt enzootic cycles and reduce human, companion, domestic animal, and wildlife exposure to infected ticks.

## 1. Introduction

Among the arthropod disease vectors, ticks transmit the greatest variety of infectious agents to companion and domestic animals, wildlife, and humans [1]. With ticks expanding their range and population sizes in response to globally changing temperature patterns [2], thereby introducing tick-borne pathogens into previously unexposed populations [3,4], a central issue is how to effectively manage, and thus prevent, the increasing public health burden posed by ticks and tick transmitted pathogens [5,6].

The effective control of ticks and tick-borne pathogens is a long standing, worldwide challenge for livestock producers and is mainly done through the use of acaricides. With the widespread resistance to different classes of acaricides as observed in multiple ixodid species [7], there exists a need for either novel acaricides that possess greater efficacy, selectivity, and less environmental impact [8], or innovative livestock tick control methods that can either be used as standalone approaches or integrated with existing methodologies. The prevention of tick-borne disease in humans traditionally relies upon personal protective behaviors to prevent tick bites, such as avoidance of tick exposure, use of repellents, tick checks to detect and remove ticks, landscape management to modify tick habitat, and environmental suppression of tick populations through the use of chemical acaricides, including those approved for use on clothing [9,10].

An effective response to these threats includes organized, large scale, federal government supported approaches to nation-wide tick and tick-borne disease control with substantial research funding, surveillance strategies, public education, and science-based policies leading to the development and implementation of new tools and products for the suppression of ticks and disease transmission, adapted to local needs and conditions [11,12,13,14,15]. The increasing awareness of the public health threat posed by ticks and tick-borne pathogens is stimulating re-evaluations of resources needed to address these problems both now and into the future. Area-wide integrated tick management approaches can greatly reduce the abundance of tick vectors and infection [16]. A commitment to greater incentives for industry and academic researchers to develop, evaluate, and commercialize novel tick and tick-borne pathogen control technologies can stimulate the development of vaccines and novel products that both repel and kill ticks [15]. Due to the interconnectedness of ticks, tick-borne pathogens, the diversity of host species, and the influence of environmental factors on tick populations, a One Health concept transformed into Ecohealth, combining an integrated, collaborative, multi-disciplinary approach among human, veterinary, and environmental health professionals, is essential for the development and implementation of effective, sustainable tick and tick-borne disease control [17,18].

As part of an integrated tick management approach, anti-tick, transmission blocking, and tick-borne pathogen reservoir targeted vaccines are recognized as promising emerging tools for advancing control efforts for ixodid and argasid ticks [19,20,21,22,23,24,25,26]. Yet, there are currently no licensed human vaccines in the United States for tick-borne pathogens [5]. Two tick-borne encephalitis virus vaccines are licensed in Europe and two vaccines for the Far-Eastern subtype of the virus are licensed in Russia [27]. Research into such vaccine strategies is showing incremental progress on multiple fronts, and continues to reveal unexpected components and pathways, as well as increasingly complex, dynamic, interconnected relationships that occur at the tick–host–pathogen interface [28]. These relationships are increasingly being explored in novel ways to develop immunological based tools to control both tick infestations and transmission of tick-borne pathogens of veterinary and human medical importance, including a diverse array of physiologically important molecules of tick tissues and saliva components, as well as organisms such as tick symbionts and the remainder tick microbiome [19,21,22,23,29,30,31,32,33,34].

**Figure 1:**
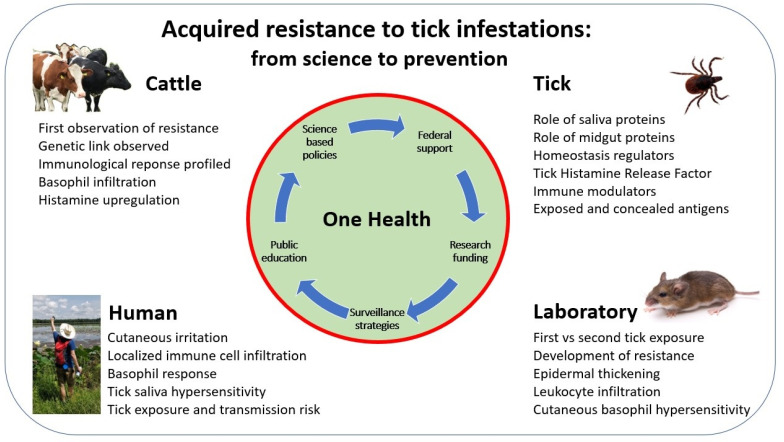
Acquired resistance to tick infestations: from science to prevention. A multidisciplinary, or One Health approach to understanding the immunological basis for acquired resistance to tick infestations. Historical data starting with the early reports on acquired resistance in cattle in 1899, to laboratory trials and human data. Our understanding of acquired resistance to tick infestations has increased substantially, both from the tick as well as from the host perspective. To bring this knowledge into successful interventions, a One Health collaborative approach between research institutions, industry, federal government, educational groups, and policy makers is required.

As research advances and new and more powerful tools and strategies provide greater resolution on a specific topic of interest, we often fail to recognize the importance of early research publications and reviews. Those reports describe the accumulating building blocks that provided the scientific underpinnings for the current understanding of the field today, and the study of the historical scope of experimental approaches and findings can provide useful information. This review examines the evolution of studies to date focused on acquired resistance to tick bites (Figure 1). Beginning with observations reported over a century ago for cattle in Australia, the progression to the current state of research to develop anti-tick and tick-borne pathogen transmission blocking vaccines is made. To develop an historical and scientific rationale for the development of anti-tick vaccines and tick-borne pathogen transmission vaccines, this review focuses on three central themes:

1. Acquired resistance to tick infestations, and the evolution of our understanding throughout decades of research.
2. Common elements of host responses leading to acquired resistance.
3. Vaccine development and the use of acquired resistance mechanisms, tick physiology, and tick microbiome as targets.

## 2. Acquired Resistance to Tick Infestations and the Evolution of Our Understanding

Ticks and tick-borne diseases negatively impact livestock production due to disease induced morbidity and mortality, impaired weight gain and milk production, and treatment costs that result in significant economic and societal consequences, particularly in the tropics and subtropics [33,35,36,37,38,39]. Lew-Tabor and Rodriguez [40] estimate that 80% of the global cattle population are at risk of tick infestation and tick transmitted infections, with a negative annual economic impact of $22–$30 billion. Considering this substantial economic impact of tick infestations in cattle, perhaps unsurprisingly, the earliest reports on tick resistance were described in cattle populations.

### 2.1. Bovine Resistance to Ticks—First Encounter

The starting point for the scientific inquiry into acquired resistance dates to the end of the 19th century (Table 1). As early as 1899, a study from Australia described that some cattle became immune to tick induced irritation in response to tick infestation in the field [41]. In a North American study, *Dermacentor* infested cattle developed tick resistance expressed by bite site exudate trapping the tick and resulting in its removal with the scab [42]. A study in Queensland, Australia, described the central elements of acquired tick resistance for tick infested cattle. Both the cattle tick and Brahmin cattle were introduced into the Northern Territory of Australia during the early 1890s, and by 1917 the economic losses caused by the cattle tick due to transmission of “tick fever”, babesiosis, leading to poor weight gain, reduced milk production, and anemia, were considered enormous. Studies performed on cattle since the 1890s revealed many of the key elements of expression of acquired tick resistance to the tick known to the authors as *Boophilus australis,* and the importance of bovine genetic composition, or breed [43]. The natural tick resistance of certain cattle breeds was reported to be an inherited attribute and was observed especially for purebred and crossbred Brahmin cattle [43]. Acquired resistance was more strongly associated with a *Bos indicus* or *Bos indicus* crossbred genetic background [44,45,46].

Johnston and Bancroft [43] attributed the induction of tick resistance to the injection of substances by the feeding tick into the bovine skin and subsequent development of bovine host antibodies that neutralized those injected substances. Bovine immunity to tick infestation research progressed along the two interrelated themes of natural, breed related resistance and acquired resistance, with the immunological basis of bovine acquired tick resistance established by Riek [44] and Roberts [47].

Subsequent studies resulted in a progressive increase in understanding the pathways, cells, and molecules involved in the acquisition and expression of the resistance response [38,48,49,50,51,52,53,54,55,56]. Low resistance to *Rhipicephalus (Boophilus) microplus* of *Bos taurus* cattle was linked to an inflammatory response at tick attachment sites that was referred to as a non-directed pathological response to infestation [50], and resistant *Bos indicus* cattle were shown to exhibit a stronger T cell and CD25+ cell response at larval attachment sites [53]. Resistant cattle were shown to have an earlier onset of cutaneous expression of proinflammatory chemokines and cytokines leading to an allergic contact hypersensitivity type response that resulted in basophil activation [54].

Acquired bovine resistance to *Rhipicephalus (Boophilus) microplus* larval infestation was associated with cutaneous allergic hypersensitivity that resulted in reduced tick engorgement weight, development of fewer adult ticks, and smaller egg masses [44]. This confirmed the initial studies by Johnston and Bancroft [43], where the defining features of acquired resistance were described to lead to a tendency toward light tick infestations, where female ticks were found to be only partially engorged, with failure to produce ova and impaired hatching of ova to the point of no viable larvae emerging [43]. The development of vesicles at attachment sites, were described as blisters, that express a lymph-like exudate that traps ticks, and ticks that fed on resistant cattle were yellow in color in addition to being undersized [43]. Hypersensitivity response at the bite site involved an influx of eosinophils and production of a serous exudate [44]. At 48 h after applying larvae to highly resistant cattle, blood histamine levels peaked and persisted for eight days, while little or no change occurred in blood histamine concentrations of infested non-resistant cattle [44]. The atypical engorgement color was subsequently shown to be due to ticks feeding on resistant hosts consuming a blood meal consisting of leukocytes rather than erythrocytes [57]. *Rhipicephalus (Boophilus) microplus* infestation was shown to result in mast cell degranulation in the skin of tick resistant cattle [58], and a histologic analysis of bovine cutaneous hypersensitivity to *Ixodes holocyclus* infestation showed an influx of basophils, eosinophils, neutrophils, and epidermal bullae formation, resulting in the trapping and killing of ticks in a serous exudate [59]. Basophil and eosinophil influxes were also observed at bite sites on resistant cattle [54]. Distinguishing features of resistant and susceptible bovine responses to *Rhipicephalus (Boophilus) microplus* infestation were reviewed in regard to inflammatory profiles, gene expression variants, and immunological determinants [38]. Skin samples from *Bos taurus* and *Bos indicus* infested with *Rhipicephalus (Boophilus) microplus* revealed CD20+ cell influxes at bite sites in both breeds and significantly increased CD3+ cells in more resistant animals [56].

Potentially underestimated as a factor in the expression of acquired tick resistance are the roles of pruritus, host grooming, and the direct effects of histamine on the feeding tick (Table 2). Histamine and its receptors are commonly associated with the sensation of itch [60,61], and antihistamines are well recognized treatments for itch [62]. Additional bioactive molecules, resulting from host innate and adaptive immune responses to ticks, mediate itch and pain responses by interacting with serotonin, Toll-like, protease activated, endothelin 1, and tumor necrosis factor receptors [63]. Infestation induced pruritus is a threat to a feeding tick and alerts the host to the presence of larvae and nymphs that feed for several days to adult females that can blood feed for over a week [64]. In cattle experimentally infested with *Rhipicephalus (Boophilus) microplus* larvae, an average of 33% engorged adults were recovered from cattle restricted from grooming, whereas adult tick recovery from unrestricted animals was only 9%. Grooming, licking, and rubbing were determined to be important mechanical responses to infestation induced pruritus resulting in tick mortality [65], and bite site cutaneous hypersensitivity reduced tick viability due to cellular reactions, licking, rubbing, scratching, and mutual grooming [66]. Further illustrating the importance of pruritus and grooming in tick resistance is the finding that cattle expressing the highest levels of resistance yielded the highest number of ticks when restricted from grooming [67]. Grooming induced tick mortality was directed primarily toward larvae within the first 24 h of infestation [67], resulting in larval losses of up to 54% [68]. The importance of pruritus was determined by studying the relationship among tick feeding, host acquired tick resistance, and itch responses for cattle infested with *Rhipicephalus (Boophilus) microplus* [44,65,66,67,69].

### 2.2. Laboratory Studies—Our Best Defense Is Knowledge

Laboratory studies of acquired resistance were logical extensions of the bovine resistance studies. The histology of tick bite sites was reported by Hoeppli and Feng [71], Trager [72], Tatchell and Moorhouse [73], and Theis and Budwiser [74]. Two foundational studies link laboratory host immune responses to tick bites. Jellison and Kohls [75] hypothesized that host immunity was responsible for poor tick feeding on rabbits repeatedly infested with adult *Dermacentor andersoni* and for the development of crust-like lesions at tick attachment sites on those rabbits. In the most frequently cited as the earliest laboratory animal study of acquired resistance, Trager [72] reported that guinea pigs developed resistance to infestation with *Dermacentor variabilis* larvae after one infestation. Acquired resistance was expressed during a second infestation as reduced tick engorgement, death of ticks, discolored feeding ticks, and small blisters at attachment sites. The histology of first exposure larval attachment sites was characterized by slight epidermal thickening with little cellular reaction, while second exposure bite sites contained large numbers of polymorphonuclear leukocytes, few eosinophils, and epidermal thickening extending below the inflammatory cell containing “mass”, and the pale color of larvae derived from the second infestation was attributed to consuming leukocytes rather than erythrocytes [72]. A seminal study by Allen [57] established that the cellular response in vesicles in hyperplastic epidermis beneath larval mouthparts on guinea pigs expressing acquired resistance to *Dermacentor andersoni* consisted of high concentrations of basophils attributed to a cutaneous basophil hypersensitivity response. During a repeated infestation in which acquired resistance was strongly expressed, the histologic changes at attachment sites consisted of epidermal acanthosis and acantholysis; dermal influx of eosinophils, lymphocytes, and macrophages with no increase in mast cells; and numerous basophils accumulating in vesicles beneath mouthparts [57]. Canines infested with *Rhipicephalus sanguineus* developed intense polymorphonuclear leukocyte infiltrations at the attachment sites accompanied by mast cell degranulation linked to tick feeding cavity formation [73,74].

The immunological basis of acquired tick resistance was strengthened by a series of studies involving the adoptive transfer of lymphocytes, passive transfer of sera, administration of immunosuppressants, and saliva driven in vitro proliferation of lymphocytes from resistant animals [76,77,78]. An in vivo examination of complement activation pathways revealed that the alternative pathway, but not the classical pathway, was needed for expression of acquired resistance [79,80]. Complement and immunoglobulins were deposited along the dermal-epidermal junction adjacent to *Dermacentor andersoni* mouthparts in resistant guinea pigs [81].

Rabbits developed and expressed acquired resistance after one infestation with uninfected *Dermacentor variabilis* adults that provided significant protection against transmission of *Francisella tularensis* type A by a subsequent infestation with infected *Dermacentor variabilis* nymphs [82]. The mechanism by which resistance to this highly virulent pathogen was expressed is not fully known. However, one possibility is that the inflammatory reaction at the tick bite site creates a milieu that reduces infectivity by containing and killing the bacteria. Guinea pigs repeatedly infested with uninfected *Ixodes scapularis* nymphs also acquired resistance that blocked transmission of *Borrelia burgdorferi* during a subsequent infestation with infected nymphs [83].

### 2.3. Human Hypersensitivity to Tick Bite—The Final Frontier

Human health threat and the impact of ticks has steadily increased since the last half of the 20th century, due to greater incidence of infections caused by established, resurging, and emerging tick-borne bacteria, viruses, and protozoa; rapidly expanding geographic ranges and populations of multiple human biting ticks; and improved diagnostics and tools to discover novel and potential pathogens associated with medically important ticks [9,14,84,85,86,87,88,89]. Tick-borne diseases are zoonoses whose enzootic cycles and human health threats are impacted by biotic and abiotic factors [90,91,92,93,94]. Geographic range expansions are occurring in North America for *Ixodes scapularis*, *Amblyomma americanum*, and *Amblyomma maculatum*, species of medical importance [9]. *Ixodes ricinus* and *Dermacentor reticulatus*, important vector species in Europe and Eurasia, are also expanding their ranges [95,96]. *Ixodes scapularis* was not considered medically important in the early 1970s; however, the discovery of its role as a vector of *Babesia microti* and subsequently of *Borrelia burgdorferi* changed that perception [9,97]. During the past thirty years, *Ixodes scapularis* was found to also be a competent vector of *Anaplasma phagocytophilum*, Powassan virus, *Ehrlichia muris*, *Borrelia miyamotoi*, and *Borrelia mayonii* [97]. *Ixodes scapularis* is of increasing importance as a vector of recognized and emerging tick-borne diseases and is expanding in geographic range [97].

The potential for the emergence of novel tick-borne pathogens is highlighted in the discovery of 24 novel viral species identified as a result of a high throughput sequencing of field collected *Amblyomma americanum*, *Dermacentor variabilis*, and *Ixodes scapularis* [98]. Currently, we know of 15 distinct tick-borne disease-causing agents infecting humans that are transmitted by eight human biting ticks in the United States, and 40% of these pathogens were described during the past two decades [9]. Lyme borreliosis is the most commonly reported vector-borne infection in the northern hemisphere with the predominant vectors being *Ixodes scapularis* in North America and *Ixodes ricinus* in Europe [99,100]. Based upon the analysis of insurance claims, approximately 476,000 patients were diagnosed and treated for Lyme borreliosis annually in the United States during 2010–2018 [101] with a significant economic burden in both Europe and North America—an estimated annual cost of up to $786 million in the United States [88].

An analysis of 25 years of biannual serosurvey data from nearly 1500 residents of an island community endemic for Lyme borreliosis revealed that individuals who experienced cutaneous hypersensitivity, itching, in response to tick bite had a significantly reduced likelihood of tick-borne disease infection [70]. Among study participants, 17% reported an itch response to tick bite during the preceding year, with tick bite induced itch incidence increasing after repeated exposures; probability of itch doubled from one to two tick bites and doubled again from two to four tick bites. The low transmission rate of *Borrelia burgdorferi* spirochetes prior to 24 to 36 h of nymphal tick feeding [102], provides a window for cutaneous irritation, itch, at the bite site to alert the infested individual. This response results in the detection and removal of the attached tick, accompanied by reduced infection risk. Indeed, the incidence of Lyme borreliosis among participants decreased incrementally with increasing episodes of tick bite associated itch events [70].

A blinded analysis of punch biopsies of *Ixodes scapularis* nymphal bite sites on human volunteers showed similar histopathology patterns in tick infested humans as in tick infested BALB/c mice. Prior tick infestations correlated directly with increased bite site accumulation of inflammatory cells, decreased vascular dilatation, and extravasation of erythrocytes, with bite site inflammation directly correlating with tick exposure [103]. As was observed in earlier studies in cattle [59] and guinea pigs [57], neutrophils, lymphocytes, and particularly eosinophils accumulated at the bite sites of tick bite sensitized individuals. Whereas the cellular changes during a first tick encounter allowed for a successful transmission of tick-borne pathogens, cutaneous hypersensitivity to tick saliva developing throughout repeated exposures led to a less permissive environment for transmission of tick-borne pathogens, with the infiltration of immune cells leading to premature tick detachment, interruption of pathogen transmission, and an opportunity for the neutralization of pathogens by infiltrating neutrophils and lymphocytes [103].

Again, similar to cattle and guinea pigs, humans were also shown to develop basophil accumulations at tick bite sites. Biopsies obtained from *Amblyomma testudinarium* attachment sites on patients showed basophil accumulation surrounding mouthparts along with neutrophils and eosinophils at 12 h post tick attachment, whereas mast cell numbers remained comparable at the mouthparts and in areas distant from the mouthparts [104]. This response pattern indicates that tick bite sites of humans would contain basophil mediators associated with pruritis as well as the emerging and diverse roles that basophils play in immune responses [105,106,107]. Cutaneous biopsies obtained from individuals naturally infested with *Ixodes ricinus* showed the development of an innate immune response at the bite site less than 24 h post tick attachment. Macrophages and dendritic cells were predominant along with elevated transcripts for chemoattractants for macrophages and neutrophils [108].

Though anti-tick and transmission blocking vaccines for use in humans are well recognized research objectives [21,23,109,110], there are very few studies of human responses to tick bites or to tick saliva molecules, despite the putative therapeutic potential of some of these molecules [111]. Translational studies are needed that focus on responses to tick feeding, specific saliva molecules, and the tick component in human host–tick–pathogen interactions.

## 3. Common Elements of Host Responses Leading to Acquired Resistance

The early observations in bovine acquired resistance and confirmation in laboratory trials and human biopsy studies showed an integral role of histamine and basophils in a successful resistance response against tick infestations. Ticks’ ability to modulate host itch, hemostasis, and immune responses is impressive, redundant, and most evident during an initial infestation for those species that develop and express acquired resistance [28,48,63,112,113,114,115,116,117]. Thus, ticks engorge readily, fully, and elicit little cutaneous irritation and inflammation during an initial infestation of species that are capable of developing acquired resistance [57,118]. However, the initial infestation stimulates the development of immune effectors that counteract actions of tick saliva components so that during subsequent infestations the actions of tick modulators of host defenses are diminished and the multiple elements of acquired resistance are expressed [28,57,63,107,112,113,114,117,118,119,120,121].

### 3.1. Basophil Response of Acquired Resistance

Both mast cell and basophil infiltrations have been documented in tick bites; several studies have shown that basophil influxes and derived mediators at tick attachment sites are integral to the phenomena of acquired host resistance to infestation (Table 3). Mast cell deficient mice successfully develop resistance to *Dermacentor variabilis* larvae following repeated infestations [122], and basophil infiltrations occur at tick attachment sites [123]. Mast cell sufficient counterparts develop a more marked resistance, suggesting a minor role for mast cells in this tick–host association [122]. In contrast to *Dermacentor variabilis* larval infestations, mast cell deficient mice of the same strain failed to develop acquired resistance to *Haemaphysalis longicornis* larvae [124]. In this tick–host relationship, it was subsequently established that histamine derived from basophils infiltrating the tick bite site, rather than from mast cells, was responsible for the expression of acquired resistance [125]. Basophil attraction to the bite was attributed to interleukin-3 produced by skin resident memory CD4+ T lymphocytes [126]. A seminal study that preserved mast cell functional integrity and selectively ablated basophils established the nonredundant role of basophils in murine acquired resistance to infestation with *Haemaphysalis longicornis* larvae [127].

### 3.2. Direct Action of Histamine on Ticks

Histamine directly impacts the expression of acquired resistance as well as mediating the itch response. Bovine acquired resistance expressed to *Rhipicephalus (Boophilus) microplus* directly correlates with bite site histamine concentration [129], and histamine and serotonin were shown to inhibit both salivation and blood uptake by *Dermacentor andersoni* females feeding on mouse membranes [130]. Histamine is a multifaceted threat to tick feeding success. Histamine concentrations at bite sites of resistant guinea pigs was increased by 500% compared to uninfested controls, and larval engorgement was reduced 92% compared to initial tick infestation. The inhibition of histamine using synthetic H1 and H2 receptor antagonists reduced the occurrence of acquired resistance [131].

A suppression of the actions of histamine is intuitively desirable during the attachment and continuous days of blood feeding by ixodid ticks, and the modulation of host histamine is an example of dynamic tick–host interactions. Tick saliva introduced during the first infestation contains molecules sufficient to reduce histamine induced itch, suppress direct histamine effects on tick feeding, and limit the inflammatory cell influx at the tick bite site. At termination of engorgement, however, histamine serves a valuable purpose for the tick to successfully detach from the host. A histamine blocker was identified in the salivary gland extract of *Rhipicephalus sanguinius sanguinius* [132], and three histamine binding proteins were detected in *Rhipicephalus appendiculatus* saliva, with each protein possessing one high and one low affinity binding site for histamine [133,134]. A histamine binding protein in *Dermacentor reticulatus* salivary glands was demonstrated to have one high affinity histamine receptor and a low histamine affinity receptor that bound serotonin with high affinity [135]. The salivary glands of the argasid tick, *Ornithodoros turicata*, express a lipocalin-like molecule with residues possessing binding affinity for histamine or serotonin [136]. A tick histamine release factor (tHRF) identified in *Ixodes scapularis* saliva was found to be an essential regulator of tick feeding, permitting successful engorgement, and recombinant tHRF was induced histamine release from basophils. Silencing of tHRF impaired tick feeding and reduced transmission of *Borrelia burgdorferi* to mice [137]. Developing an effective suppression of histamine and serotonin appears to be a common evolutionary event across tick families.

### 3.3. Tick Saliva: Many Questions Remain

Unraveling the complexities of tick saliva remains a major barrier to achieving an understanding of tick–host pathogen relationships and the development of saliva molecule-based infestation and disease transmission control strategies. A significant gap in our knowledge is definitively identifying the specific saliva molecules responsible for the modulation of host defenses of itch, hemostasis, immunity, and wound healing. Understanding the complexity, redundancy, diversity, differential expression, and interspecies and intraspecies variations of tick salivary gland derived molecules increased dramatically due to the application of transcriptomics, next generation sequencing, quantitative proteomics, and increasingly powerful bioinformatics tools [28,48,63,107,112,113,114,115,116,117,120,121,128,138,139,140,141]. Becoming increasingly clear, while simultaneously more complex, are the ways tick-borne infectious agents exploit tick saliva modulation of host defenses to create environments favorable for pathogen transmission and establishment [28,113,114,116,119,121,142].

The selection of tick saliva molecules for use in anti-tick vaccines is still, at best, an educated guess. The greatest challenge to advancing multiple areas of tick–host–pathogen research is linking individual molecules to specific biological activities at the tick–host–pathogen interface. If saliva molecules are to be incorporated into anti-tick vaccines, the identification of biological activities of specific molecules, gene families, and redundant molecules needs to be achieved. The challenges of identifying protective saliva gland antigens are increased due to salivary gland gene differences between and within prostriate and metastriate tick species; gene transcription changes during infection with tick-borne pathogens; broadly conserved multigenic families; plurapotency and redundancies of gene products that target specific host defenses; and, saliva composition that changes during the course of feeding, including within members of a gene family [111,115,143,144,145,146,147,148,149]. The complexity of these relationships is made more challenging to unravel by the adaptation of salivary gland gene expression in response to species of the infested host [150]. While proteins have been the predominant focus, the tick salivary gland also produce non-protein compounds with established biological activities [151]. Salivary gland transcriptome analysis revealed differences for *Rhipicephalus (Boophilus) microplus* feeding on susceptible or resistant hosts that resulted in the recommendation that stress response genes are potential targets for tick control during blood feeding [152].

## 4. Anti-Tick Vaccination Strategies

Immunization was recognized early on as a feasible strategy to be developed for control against ticks and has been a research objective for over eighty years. Guinea pigs administered an intracutaneous inoculation of an extract of *Dermacentor variabilis* larvae developed an immune response that inhibited 66 to 100% of a subsequent larval challenge [153]. An important observation was that guinea pigs developed antibodies when immunized with digestive tract and salivary glands of partially fed female *Dermacentor variabilis* and when immunized with salivary glands derived from unfed females [153]. Reports that followed over the next few decades focused primarily on the use of tick tissue extracts, especially salivary glands and digestive tracts [48,112,138,154,155,156,157].

The diversity and specificity of tick tissues, molecules, and strategies used in anti-tick vaccine research increased greatly over the years based upon successive advances in the understanding of tick physiological pathways, cell biology, genomics, functional genomics, and quantitative proteomics. The challenge is identification of tick antigens capable of inducing a consistently highly protective anti-tick response that significantly disrupts tick feeding, inhibits direct infestation damage, and blocks pathogen transmission. The majority of anti-tick immunization trials performed to this day have resulted in variable levels of protection that likely would not sufficiently reduce tick infestations to eliminate damage due to tick feeding and pathogen transmission [23,29,30]. While representing potentially very useful tools to add to integrated tick management systems, the development and implementation of successful anti-tick vaccines requires the further identification of immunogens central to maintaining tick homeostasis [158].

### 4.1. The Use of Acquired Resistance Mechanisms as a Guide for the Development of Vaccines

Cutaneous itch, hypersensitivity, is a common component of acquired resistance to ticks across diverse host species. Basophils and mediators released from them appear to be important effectors that alert the host to tick infestation and create an environment at the tick attachment site that is deleterious to successful blood feeding and pathogen transmission. Whether the successful use of tick saliva molecules to induce protective anti-tick immunity requires the expression of bite site hypersensitivity and subsequent tick removal behaviors, remains an open question. Vaccines could be designed to elicit a basophil response at tick attachment sites and structured in such a way to induce an immune response at the bite site, alerting the host to the tick’s presence. However, while the elicitation of such a cutaneous hypersensitivity response may be an option for livestock and wildlife vaccines, it would likely be far less tolerated for anti-tick and transmission blocking vaccines for human and companion animal use.

Immunization induced host protection against tick infestation has been a research objective for over eighty years. Guinea pigs administered an intracutaneous inoculation of an extract of *Dermacentor variabilis* larvae developed an immune response that inhibited 66 to 100% of a subsequent larval challenge [72]. An important finding was that guinea pigs developed antibodies when immunized with digestive tract and salivary glands of partially fed female *Dermacentor variabilis* and with salivary glands derived from unfed females [153]. The diversity of tick tissues, molecules, and novel strategies used in anti-tick vaccine research increased greatly over the years with each successive advance in understanding tick physiological pathways, cell biology, genomics, and functional genomics.

### 4.2. Host Antibodies Enter Tick Haemocoele: More Potential Vaccine Antigen Targets

Although available at times as different constructs in different regions of the world, effective modern and widely accessible vaccines remain a goal for the control of four major tick-borne diseases of livestock: anaplasmosis, babesiosis, heartwater (formerly cowdriosis), and theileriosis [159,160,161,162].

Large molecules can move across the digestive tracts of blood feeding insects and ticks into the hemolymph, where they can retain their biological reactivity, and interact with internal tissues. This finding has important implications for antigen selection and vaccination strategies to limit blood feeding arthropods. Host antibodies, obtained from rabbits immunized with fly tissues, retain their biological activity in the blood meal of *Sarcophaga falculata*, and pass across the digestive tract into the hemolymph, where they bind to the corresponding immunizing tissue [163]. During a period of approximately two hours, argasid ticks consume a much smaller blood meal than ixodid larvae and nymphs that may complete a blood meal in four days or adults during potentially more than a week [64,164]. Highly relevant to immunizations with internal tick tissue were the observations that host immunoglobulins consumed in the tick blood meal passed serologically intact across tick gut into the hemolymph and were subsequently present in a salivary gland extract [165,166]. Immunofluorescent microscopy confirmed that rabbit antibodies raised against tick ovaries and salivary glands, when consumed in a blood meal, retained tissue binding specificity in *Dermacentor variabilis* hemolymph [165]. Likewise, hemolysins raised in rabbits to sheep erythrocytes retained their antigen binding ability in the hemolymph of female *Ixodes ricinus* [167]. Intact rabbit immunoglobulin G was also present, post-blood meal, in hemolymph of the argasid tick, *Ornithodoros moubata* [168]. A comparative study of seven species showed that highest concentrations of intact immunoglobulin G (30%) was found in *Hyalomma excavatum*; total immunoglobulin concentrations in *Ornithodoros moubata* were comparatively low; and 100% of the antibody molecules were intact [169]. Functional bovine antibodies persisted in *Rhipicephalus (Boophilus) microplus* hemolymph for at least 48 h post-engorgement [170], allowing for binding to target antigens that might be undergoing differential expression during tick feeding.

Hemolymph immunoglobulin G antibody specific activity was 35 to 42% for *Rhipicephalus appendiculatus* females that fed upon guinea pigs immunized with killed *Escherichia coli* [166]. Central to understanding transport into the tick hemocoel was the observation that immunoglobulin G molecule Fc piece was identified as the region essential for specific uptake across the *Amblyomma americanum* midgut into hemolymph, and receptor mediated endocytosis was speculated to be the mechanism for the preferential transport of immunoglobin G from midgut to hemolymph [171].

Considering the ability of host immunoglobulins in the blood meal to retain their binding specificity upon transport into the hemolymph, anti-tick vaccines focused on tick physiology are an area of increasing interest. Research focused on the identification of relevant target antigens and mechanisms to achieve functionally high enough host antibody titers in the hemolymph of the engorging tick to disrupt normal function or cause tick death, is warranted. The immunological disruption of tick digestive tract integrity should be explored to enhance entrance of host antibodies into the hemolymph combined with antibodies targeting physiologically critical molecules on tick tissues bathed in hemolymph. There appears to be a large pool of potential antigens, and for the successful development of such vaccines, continued advances in understanding the physiology, biochemistry, cell biology, receptor repertoires, and mediators of tick digestive tract, reproductive tissues, nervous system, water balance, excretion, heme processing, and salivary gland function, are required.

### 4.3. Tick Tissue Antigens

A foundational study demonstrated that *Dermacentor andersoni* midgut and reproductive organs were sources of protection inducing anti-tick vaccine antigens [172]. Subsequent elegant studies in Australia advanced from tissue extract immunizations and the identification of induced immune response mediated damage to establishment of the most significant proof of concept for anti-tick immunization and commercialization, using the *Rhiphicephalus microplus* gut glycoprotein Bm86 [173,174]. Anti-Bm86 IgG antibodies act by binding digest cells, interacting with host complement, inhibiting blood meal endocytosis, and damage to the gut resulting in tick death and inhibited ovaposition [174]. Homologues of Bm86 were identified in both *Rhipicephalus annulatus* and *Hyalomma anatolicum anatolicum* [175,176]. As *Rhipicephalus microplus* resistant to anti-Bm86 emerged, Bm95 was identified as a vaccine antigen that was effective against both Bm86 resistant and susceptible strains [177]. A tick derived ribosomal 20 amino acid peptide, P0, when conjugated to Bm86 induced antibody responses that were protective at 86% and 84% efficacy, respectively, for canines challenged with *Rhipicephalus sanguineus* and cattle infested with *Rhipicephalus microplus* [178]. A structurally related protein, ATAQ, with multiple epidermal growth factor like domains was identified in the midgut and Malpighian tubules of all life stages of *Rhipicephalus microplus* and *Rhipicephalus appendiculatus* [179], however, only modest reduction in tick performance was observed when ATAQ was assessed as an anti-tick vaccine [180].

We are reminded that the host immune effectors induced by immunization extend beyond antibodies. The immunization of calves with whole *Ixodes ricinus* and midgut extracts resulted in hyperemia and exudative blisters at attachment sites accompanied by significantly fewer ticks successfully feeding [181]. In vitro feeding experiments revealed that antibodies derived from extract vaccinated calves were not the only immune effectors responsible for the observed anti-tick immunity [181].

Physiologically critical molecules are important targets for anti-tick vaccines. Aquaporins are molecularly well characterized integral membrane channels essential for water transport [182] in vertebrate and invertebrate species [183]. The ability to maintain water balance is a critical issue for a tick consuming blood and salivating into the bite site over a period of days to more than a week [64]. Much of the water consumed in the tick blood meal is transported across the midgut to the salivary glands and secreted back into the host [64,184,185,186]. An aquaporin identified in *Ixodes ricinus* is abundant in gut, rectal sac, and salivary glands, tissues critical in mass movement of water [183]. The disruption of tick osmoregulation by inhibiting aquaporin function has the potential to rapidly inhibit tick feeding, disrupt molting, cause tick death, and inhibit pathogen transmission. The silencing of *Rhipicephalus microplus* aquaporin functionality established the critical role of these molecules in successful blood feeding [187]. Cattle vaccinated with a recombinant epitope of a *Rhipicephalus microplus* aquaporin elicited a specific immune response that significantly reduced the number of feeding ticks by 68% and 75% in two respective trials [188]. Additional studies employing immunization with aquaporin derived from the cattle tick, *Rhipicephalus microplus*, induced cross-protective immunity in dogs against *Rhipicephalus sanguineus* [189]. In another study, aquaporin vaccination induced resistance to *Ixodes ricinus* larvae, resulting in an 80% reduction in tick molting and survival [190].

The vaccine potential of tick aquaporins is reflected by in silico analyses to identify tick specific aquaporin-1 motifs for potential anti-tick vaccine applications [191]. Few studies addressed host immunization to disrupt argasid tick infestation. Midgut endothelial cell proteins of *Ornithodoros erraticus* used as an extract to immunize pigs and mice induced protective responses that resulted in mortality of up to 80% of a nymph challenge and reduced female fecundity by 50% [192]. Midgut extract fractionation identified a luminal surface protein, Oe45, to which much of the protective response induction was attributed [192]. Midgut transcriptomic and proteomic analysis were used to identify two aquaporin vaccine candidates for the argasid tick, *Ornithodoros erraticus* [193]. Aquaporin immunized rabbits developed strong antibody responses and upon challenge with adults and nymphs resulted in a significant reduction in adult female fertility [193]. Based upon the findings of these 2007 and 2019 studies, there appear to be multiple midgut antigens that in combination induce a robust protective immune response to *Ornithodoros erraticus*. Argasid ticks are an important topic for anti-tick vaccine development due to the importance of several argasid species in transmission of African swine fever and the important role of feral swine in maintaining the transmission cycle of this devastating virus [194].

Subolesin is a regulatory protein of the tick innate immune response that is involved in the downstream induction of signal transduction pathway elements in response to infection [195]. Artigas-Jerónimo et al. [196] reviewed the structure, function, evolution, and potential utility as vaccine antigens of subolesin/akirin, widely conserved proteins with diverse biological roles across metazoan species. Subolesin remains a major focus of interest for development of anti-tick vaccines, including vaccines consisting of multiple antigens and anti-tick combined with anti-pathogen formulations.

A multiple antigen anti-tick vaccine construct of recombinant subolesin, attachment cement protein TRP64, and three histamine binding proteins was combined with an anti-*Theileria parva* sporozoite antigen to vaccinate cattle that were subsequently infested with *Rhipicephalus appendiculatus* nymphs, adults and an infected tick challenge with *Theileria parva* [197]. Each antigen elicited a strong immune response. However, the vaccination regimen did not impair tick feeding or *Theileria parva* transmission [197].

In vitro feeding was used to assess the combined effects of antibodies reactive with Bm86 and subolesin on larval engorgement of *Rhipicephalus australis* [198]. Larvae that fed upon bovine anti-Bm86 serum were reduced by 39%; however, that decrease was not statistically significant compared to control serum fed larvae. Anti-subolesin antisera had no effect on larval feeding. A synergistic effect was observed by combining anti-Bm86 and anti-subolesin antibodies. Feeding of larvae fed equal volumes of the two antisera were reduced by 63% compared with control sera fed larvae [198]. A multiple antigen vaccination regimen induced up to 87% efficacy, based upon assessing tick mortality, reduced blood meal and reduced reproduction of *Haemaphysalis longicornis*, resulting from an initial DNA vaccination expressing subolesin followed by a booster injection of a subolesin polypeptide of a chimeric subolesin-PO polypeptide [199]. Kasaija et al. [200] highlighted important considerations for anti-tick vaccine development when assessing vaccination with subolesin to induce protection against multiple tick species by livestock within a specific geographic region. Parameters to consider include sequence variations within antigens of interest and the genetic, immune, and physiological backgrounds of livestock populations to be protected.

### 4.4. Salivary Gland Derived Antigens

Tick saliva inhibition of hemostasis is an established phenomenon [201,202]. Anti-tick vaccine candidates include tick saliva inhibitors of hemostasis [203]. Three *Amblyomma sculptum* protease inhibitors were characterized as inhibitors of factor Xa, trypsin, and/or thrombin, assessed as vaccine antigens, and found to induce variable levels of protection against adult female and nymph challenges [204]. All three proteins inhibited an activation of both the alternative and classical pathways of human complement. Vaccination efficiency against adult females ranged from 59.4% to 85% and mortality of nymphs fed upon vaccinated mice ranged from 70% to 100% [204].

Cattle immunized with a recombinant *Rhipicephalus microplus* salivary gland metalloprotease, designated rBrRm-MP4, upon challenge infestation were afforded 60% protection expressed as increased tick mortality, impaired oviposition and reduced egg hatching [205].

Attachment cements are protein salivary gland secretions produced early in the process of mouthpart insertion by members of the Ixodidae to anchor the feeding tick to the host and to seal the space between the mouthparts and host skin [206]. A 15 kDa *Rhipicephalus appendiculatus* attachment cement protein, 64P, was cloned, expressed, and used to induce host immunity to multiple tick species that was expressed as reduced attachment and feeding and death of engorged ticks due to cross reactivity with midgut antigen [21,202]. Four truncated versions of 64P were constructed for the vaccine in order to expose and enhance the host response to multiple antigenic regions. Incorporating 64P as an antigen, a multiple antigen anti-tick vaccine was constructed by combining recombinant subolesin with a trio of different histamine-binding proteins, TRP 64 attachment cement protein, and p67C *Theileria parva* sporozoite antigen [197]. Immunized cattle developed robust immune responses to each antigen. Cattle were challenged with two strains of *Rhipicephalus appendiculatus* to assess anti-tick immunity and transmission of *Theileria parva*. No correlations were established between bovine immune responses and tick feeding parameters with females from vaccinated cattle producing a higher egg mass weight than ticks that fed upon controls [197].

Ixodes scapularis saliva protein Salp15 was extensively studied due to its biological activities of host immunosuppression and protection of *Borrelia burgdorferi* from antibody attack [207,208]. Host T lymphocyte proliferation and activation are reduced by Salp15 inhibition of the activating cytokine interleukin-2, IL-2; impairment of T lymphocyte signal transduction; and the suppression of dendritic cell proinflammatory cytokines and activation [207,209]. The T lymphocyte surface receptor for Salp15 is helper cell expressed CD4 [210]. *Borrelia burgdorferi* outer surface protein C, OspC, binds Salp15 that protects the spirochete from antibody mediated lysis [211]. Anti-Salp15 antibodies interfere with Salp15 binding to OspC, resulting in enhanced antibody mediated, complement dependent killing of *Borrelia burgdorferi* [212]. Adenovirus vectored Salps, including Salp15, combined with *Ixodes scapularis* Isac, an inhibitor of alternative complement pathway C3 convertase [213], reduced *Borrelia burgdorferi* burden in infected mice by 60% [214]. Immunization of mice with Salp15 protected 40% of vaccinated mice from *Borrelia burgdorferi* infection while 95 to 100% of control mice were spirochete positive following infected tick challenge, indicating a protective potential [215].

Modest cross protection was observed when rabbits immunized with *Rhipicephalus microplus* cystatin 2c slightly reduced the number of engorging females by 11.5%, adult female engorgement weight by 5.8%, and resulted in damaged gut, salivary glands and ovaries of *Rhipicephalus appendiculatus* [216]. Glutathione S-transferase cloned from *Dermacentor marginatus* used as an antigen induced rabbit immune responses that resulted in approximately and overall 44 % reduction in female tick engorgement, egg mass, and hatching [217].

An illustration of the gaps in our knowledge is that not all vaccinations negatively impact tick parameters. For example, enhanced engorgement and molting accompanied by decreased mortality were unexpected findings after immunization with an *Ixodes ricinus* serine protease inhibitor and a lipocalin [218].

### 4.5. Novel Antigen Sources

Relatively recent advances in defining tick microbiomes are yielding multiple new insights into tick physiology, reproduction, production of essential molecules not synthesized by the tick, tick-borne pathogen-vector interactions, and a potential new avenue for development of anti-tick vaccines [219,220,221,222,223]. Metagenomic analyses revealed considerable complexity of bacterial genera in life cycle stages and geographical variations in bacterial community structure that indicates a role for environmental influences [224,225]. Tick-borne pathogen colonization is influenced by the action of microbiome constituents on the midgut epithelial barrier [226]. Microbiome-tick communication occurs in both directions as illustrated by an *Ixodes scapularis* gut secreted protein that facilitates colonization with *Borrelia burgdorferi* [227]. Symbiont and commensal bacteria influence both tick colonization by and transmission of tick transmitted pathogens [223].

Tick microbiome was proposed as a target for biocontrol of *Ixodes scapularis*, when it was observed that bacterial species isolated from nymphs were qualitatively different from those cultured from adults [228]. Subsequently, microbiome complexity and variations discovered by metagenomic analyses provided potential targets for disruption of essential biological processes within the tick. Could tick microbiome species be suitable targets for vaccination-based control? Anti-tick microbiome vaccination rationale is that tick and pathogen transmission control can be achieved by disruption of bacterial species that are central to the support of other microbiome species and essential tick physiological functions [34]. An innovative study revealed that host antibodies, induced by immunization with a tick microbiome Enterobacteriaceae, caused significant mortality of engorging ticks [34]. Mean tick mortality was approximately 45% in the target immunization group. That level of vaccine induced protection is consistent with anti-tick vaccines that incorporated secreted, membrane associated, or intracellular protein antigens [23,29,229]. Anti-tick vaccine directed at the microbiome of *Ixodes scapularis* disrupted both the makeup and functions of the microbiome and increased pathways central to biofilm formation, a possible microbiome defensive response [230]. The effectiveness of anti-tick microbiome vaccination strategies will be refined based upon further resolution of microbiome constituent species and their functions. Vaccine antigens could include multiple tick microbiome symbionts and combinations of anti-microbiome antigens with molecular constituents of tick physiological pathways integral to maintaining homeostasis.

Cell free hemolymphs derived from fully engorged female *Amblyomma americanum* and *Dermacentor variabilis* were used to immunize rabbits that were subsequently infested with larvae, nymphs, and adult of the homologous tick species [231]. No statistically significant difference was determined between immunized and control groups for engorgement weights of nymphs, females, and egg masses. The absence of statistical significance was attributed to large standard deviations around the treatment group means [231]. Although not successful, the use of signaling molecules in tick hemolymph is an approach that merits further consideration. The selection of hemolymph from fully engorged female ticks in the Ben-Yakir and Barker [231] study might not be optimal for the identification of optimal vaccine targets. Knowledge about hemolymph components is still relatively rudimentary; however, it has advanced significantly [232]. Tick hemolymph composition is high in proteins, carbohydrates, amino acids, and lipids compared to vertebrate blood [233]. The spectrum of hemolymph molecules includes vitellogenins, antimicrobial peptides, heme binding carrier proteins, lectins, proteases, protease inhibitors, macroglobulins and additional physiologically important molecules [232].

Use of hemolymph molecules as potential control targets is receiving increased attention. Immunoglobulin binding proteins in tick hemolymph are important for movement of these large proteins to the salivary glands for excretion and immunization to disrupt these proteins is thought to be a means to enhance effectiveness of host antibodies that react with tick internal tissues [234]. Rabbits vaccinated with a serine protease inhibitor found in hemolymph of *Haemaphysalis longicornis* resulted in mortality of 45% and 43% of challenge infestation nymphs and adults, respectively [235]. Another novel vaccine target is the vitellogenin receptor that is important in oocyte yolk formation by binding vitellogenin in the hemolymph to initiate endocytosis into the egg and conversion to vitelline, a critical nutritional source for tick embryos [236]. Tick iron and heme metabolism proteins are potential anti-vaccine targets to disrupt tick metabolism of these potentially toxic molecules in the blood meal [237]. Ferritin based anti-tick vaccines did not induce bovine resistance to *Ixodes ricinus* infestation [181] but did stimulate a significant drop in engorgement weights in adult *Haemaphysalis longicornis* infesting immunized rabbits [238]. Tick innate immune defense signaling pathways, soluble molecules, hemocytes, and complement-like molecules are an additional complex of targets for anti-tick and tick-borne pathogen transmission blocking vaccines [239].

A range of different internal tick antigens have been considered as anti-tick vaccine antigens. An interesting vaccination trial antigen choice was a cysteine-rich protein associated with muscle development in *Haemaphysalis longicornis* that provided minimal protection with 20% adult challenge mortality and a 17.4% decrease in engorgement weights [240].

The diversity and number of anti-tick vaccine potential antigens are increasing (Table 4). There exists a growing consensus that a successful anti-tick vaccine will likely be comprised of multiple antigens that are cross-reactive and thus cross-protective [241]. The development of a vaccine consisting of multiple antigens is appealing, an example being use of exposed, such as soluble protein, and concealed, membrane component, antigens as proposed by Trimnell et al. [242]. In light of the increased interest in antigen cocktail anti-tick vaccines, Ndawula and Tabor [22] examined potential limitations and strategies for enhancing multiple antigen vaccine efficacies.

## 5. Reservoir Targeted Vaccines

A large body of research has focused on the development of anti-tick and pathogen-transmission blocking vaccines for use in livestock and companion animals. No such vaccines have made it into the human health space yet. With many tick species spending (at least) a portion of their life cycles in a wildlife reservoir environment, and many studies being initially performed in laboratory mice, the potential of using murine wildlife reservoirs as antibody factories for reducing tick numbers and pathogen colonization in the tick provides novel ways to reduce ticks and tick-borne diseases.

Ecological studies on the impact of wildlife vaccination and the intricate relationship between different wildlife populations, or systems ecology, can guide such vaccination strategies. Mathematical modeling plays an important role in defining the impact wildlife interventions could have on an established ecosystem. A study on the environmental measures for the control of tick abundance and reduction of Lyme disease in New Jersey, analyzed the effects of burning of shrubbery, reduction of reservoir availability, and dispersion of chemicals on ixodid tick abundance [243]. Whereas reduction of deer and mice populations is not always politically or ecologically advised, and the use of chemicals is increasingly being prohibited, all measures indicated a substantial decrease of tick abundance. This decrease, however, only lasted in the year the measurements were applied, indicating that tick abundance is very resilient and heavily regulated by the tick population [243]. A model looking at the population of murine reservoirs in relation to ixodid tick abundance in Siberia showed that there is a threshold level below which murine populations are no longer able to support tick populations and could aid in the reduction of ticks [244]. In addition to having reservoir populations too low to support tick populations, a model taking into account transmission of tick-borne pathogens showed that when reservoir populations become too high, disease transmission can be hindered due to a dilutive effect [245]. Using dynamic population models considering the different life stages and environmental factors, it was shown that the tick system exhibits complex behavior with an unstable equilibrium [246,247]. Indeed, modeling used to predict the effect of host immune systems on tick populations showed that immune reactions in the host can decrease the size of tick population equilibrium, thereby reducing the risk of transmission of tick-borne diseases [248]. In contrast, research on biodiversity and zoonotic disease risk has shown that the loss of biodiversity can increase the risk [249,250]. Taking away a natural reservoir such as the mouse, for example, increases the likelihood that an incidental host, such as a human, gets in contact with an infected tick. The results of these modeling efforts give valuable information on how to control tick-borne diseases and make a strong argument for vaccinating wildlife reservoirs, rather than just eliminating them. This raises an interesting question, that if we were to vaccinate enough reservoir species to mimic the effect of having too many reservoir animals per infected tick, could we observe a similar effect, without increasing the risk of tick encounter by eliminating this wildlife reservoir? Further development of these models, and validation using field trials testing for the impact of the intervention not only on the tick and/or pathogens, but also on the ecosystem, is warranted to further optimize these models to be able to use them in a predictive analytics framework.

In order for a reservoir targeted vaccine to successfully reach the target population, a number of additional considerations come into play. Unlike domesticated animals and humans, vaccines need to be formulated and dispersed in such a way that wild animals come into contact with the vaccine. This bait needs to be attractive to entice consumption, able to survive weather conditions, not interfere with the natural foraging behavior of the animals, and plentiful to allow for repeated exposures [25,26,251]. Oral vaccination using vaccine-laced bait has been explored for use in murine populations, and numerous field trials have been performed using the Borrelia Outer Surface Protein A (OspA) for vaccination of wild *Peromyscus leucopus* mice and show a reduction in the number of ixodid ticks positive for *B. burgdorferi* spirochetes [24,252,253,254,255,256,257,258].

With these successful studies using OspA for vaccination of wild *P. leucopus* mice, now is the time to start examining the potential of the existing and newly described antigens to be successful in a reservoir targeted vaccination setting. Some of the limitations described for the vaccination of livestock, companion animals, and humans could be of less concern when vaccinating a wildlife population that could potentially offer higher tolerance to potential unwanted side effects. While grooming behaviors in cattle populations resulted in the loss of in particular larval infestations [67,68], an induction of grooming can also lead to decreased hide quality and additional risks in these cattle. However, in a wildlife, especially murine, population with a much shorter lifespan, this could prove to be less of a concern, and the induction of cutaneous hypersensitivity, basophil recruitment, and histamine modulators counteracting the tick histamine release factor could effectively reduce tick infestation and pathogen transmission in these wildlife populations. Studies focusing on these types of vaccines will need to be validated in the wild where continuous exposure to ticks and other irritants can provide a natural enhancement of efficacy and potential adverse effects can be studied in their natural environment. 

Perhaps the largest limitation that has been observed throughout in the development of anti-tick vaccines is the sub-optimal efficacy, possibly, at least partly, due to the redundancy and therefore compensatory action of tick molecules [28,48,63,112,113,114,115,116,117]. A vaccine indicated for use in livestock, companion animals, and humans alike, will have to show noticeable and rapid reduction in tick infestation, with the desired effect being equal or better than the use of acaricides. This is important both in adoption of the vaccine (“perceived efficacy”), as well as in ensuring pathogen transmission risk is markedly reduced, if not eliminated (“absolute efficacy”). The Bm86 vaccine, as currently used in livestock for reduction of *Rhipicepahlus (Boophilus) microplus* infestations, perfectly illustrates this with efficacy shown to be >80% in cattle and canines [173,174,178]. The success of this vaccine also makes a case for the use of vaccines targeting physiologically critical molecules, leading to the reduction of successful tick feeding and ovaposition. For use in wild life, the requirements for such a vaccine could be less stringent, where reduction of ovaposition and cumulative reduction of tick populations could lead to the desired effect in subsequent populations. Increasing the number of tick-resistant mice in the wild, would mimic a situation where less mice are available for ticks to sustain their population equilibrium without affecting the ecology, as the mouse population stays constant, but the tick population is decreased. 

The observation that antibodies can successfully pass through the tick hemolymph [165], clearly offers an opportunity for reducing or eliminating the pathogen abundance through a reservoir targeted vaccination strategy. A successful wildlife vaccine would ideally eliminate pathogens at the tick level and/or reduce successful tick propagation, opening an opportunity for vaccines targeted at pathogen specific and/or tick gut and hemolymph antigens, allowing for longer attachment while still having an impact on (infected) tick populations and pathogen abundancy. Impact of such vaccines is shown through vaccination of mice with the OspA vaccine where only 17% of infected ticks placed on vaccinated animals were still positive for Borrelia spirochetes in culture [254], and vaccination strategies targeting Salp15 in mice, which showed complement dependent killing of *Borrelia burgdorferi* through binding with the Borrelia burgdorferi OspC [212]. 

With the roles of tick physiology and microbiome becoming ever clearer with the availability of new techniques, combination strategies targeting tick gut health and inhibiting pathogen replication are of interest. The reported 45% tick mortality using vaccination against tick microbiome Enterobacteriaceae [34], and 45% nymphal mortality using serine protease inhibitors vaccines [235] may be deemed suboptimal for use in livestock, but their use in wildlife population, possibly combined with pathogen specific vaccines, should not be dismissed as opportunities for disrupting tick population equilibria and creating a hostile environment for tick-borne pathogens. 

In the development of anti-tick and pathogen transmission blocking reservoir targeted vaccines, the criteria for success will depend not only on the direct effect on a single host or tick, but the cumulative effect observed in subsequent generations of both host and tick. Multi-year field trials in combination with mathematical modeling can aid in developing specific criteria to accurately set expectations for a successful reservoir targeted wildlife vaccine.

## 6. Concluding Thoughts

The development of effective anti-tick and transmission blocking vaccines requires an in depth understanding of the genetics, cell biology, immunology, and pharmacology of acquired resistance to tick infestation as well as tick countermeasures to host defenses, tick physiology, and tick salivary secretions. Adding to the challenges of vaccine development are the temporal biochemical and physiological changes that occur in both ticks and hosts throughout blood feeding, which can last for multiple days, and the evolutionary conservancy, diversity, and redundancy of response patterns at the tick–host interface. These are daunting tasks. Significant gaps exist in our knowledge with some essential aspects less well defined than others. Successfully addressing those knowledge gaps is a factor for continuing to make more rational “best assessments” as to optimal protection inducing antigens and the best strategies by which to stimulate and sustain host immunity to tick bites and tick transmission of pathogens.

Although significant advances are occurring in salivary gland transcriptome and proteome analyses, linking specific molecules, individually or in combination, to distinct biological functions at the tick–host interface remains to be achieved. These unknowns are an impediment to development of saliva focused vaccine strategies. Likewise, saliva component temporal expression profiles, redundancies and variations driven by host selection and across tick species distributions are largely unexplored. With acquired resistance being characterized already in the early 1900s and the role of basophils, histamine, and the cutaneous itch response still revealing new facets of the intricate tick–host interplay, the pertinent question is whether this natural response can be exploited in the development of vaccines. Stimulation of grooming responses could be beneficial in livestock and wildlife but would be less so when used in humans or companion animals. Not only the similarities, but also the differences between acquired resistance responses between both tick and host species need to be taken into account, and perhaps successful vaccination strategies need to be tailored specifically to either the tick, host, or both.

With geographical expansion of ticks and tick-borne diseases, now is the time to combine immunological, ecological, and veterinary forces. The past few years have seen a tremendous increase in the microbiology toolset, with genomics, transcriptomics, and proteomics being widely available. Applying these tools to fully characterize the tick and host throughout tick feeding, could lead to a better understanding of the intricate tick–host interplay and lead to successful vaccine strategies.

## Figures and Tables

**Table 1:** Major early observations about bovine resistance to tick infestations.

Observations	Publication	Reference Number
Cattle develop immunity to tick infestation in field	Hunt, 1899	[41]
Cattle resistance developed to *Dermacentor andersoni* infestation associated with exudate that traps ticks	Bishopp and Wood, 1913	[42]
Expression of bovine acquired resistance to tick infestation has a genetic component, especially for *Bos indicus* background	Johnston and Bancroft, 1918; Riek, 1962	[43] [44]

**Table 2:** Cutaneous hypersensitivity and host grooming response to reduce tick infestation.

Cutaneous Hypersensitivity Induced Grooming Response Observations	Publication	Reference Number
Bovine acquired resistance cutaneous hypersensitivity associated with exudate that traps ticks, bite site vesicles, poorly fed and dead ticks	Bishopp and Wood, 1913; Johnston and Bancroft, 1918; Riek, 1962	[42] [43] [44]
Increased histamine levels; Mast cell degranulation at bite sites on resistant cattle; Basophil influx with ticks trapped in serous exudate	Riek, 1962 Schleger et al., 1976 Allen et al., 1977	[44] [58] [59]
Grooming in response to tick induced pruritus is an important factor in expression of acquired resistance	Snowball, 1956 Bennett, 1969 de Castro and Newson, 1993 Hart, 2000	[65] [67] [66] [69]
Human hypersensitivity to tick bite correlates with reduced incidence of tick-borne infections	Burke et al., 2005	[70]

**Table 3:** Basophils as key cells in expression of acquired tick resistance.

Observations	Publication	Reference Number
Basophils in acquired resistance to ticks	Karasuyama et al., 2018, 2020; Yoshikawa et al., 2021	[107,120] [128]
Basophil and mast cell similarities and differences in their biology, roles in host defense and disease pathogenesis, and availability of specific molecular tools to distinguish their effector functions	Voehringer, 2013; Karasuyama et al., 2018, 2020 Tabakawa et al., 2018 Yoshikawa et al., 2021	[106] [107,120] [125] [128]
Basophil function as antigen presenting cells for Th2 responses	Ohta et al., 2017 Karasuyama et al., 2018, 2020	[126] [107,120]

**Table 4:** Selected examples of anti-tick vaccination antigens assessed.

Antigen Category	Tick Species	Antigen Types	References
Midgut, Reproductive Tissue, Malpighian Tubule			
	*Dermacentor andersoni*	Extracts of midgut and reproductive tissues	[172]
	*Rhipicephalus (Boophilus) microplus*	Bm 86, BM 86 combined with Bm 95	[173,174,177]
	*Rhipicephalus (Boophilus) annulatus*, *Hyalomma anatolicum anatolicum*	Bm 86 homologues	[175,176]
	*Rhipicephalus sanguineus,* *Rhipicephalus (B.) microplus*	Ribosomal peptide, PO, combined with Bm 86	[178]
	*Rhipicephalus (B.) microplus.* *Rhipicephalus appendiculatus*	Midgut and Malpighian tubule protein with epidermal growth factor domains	[179]
Water Balance			
	*Ixodes ricinus*, *Rhipicephalus (B.) microplus*	Aquaporins with essential roles in blood feeding	[183,187]
	*Rhipicephalus (B.) microplus*, *Rhipicephalus sanguineus*, *Ixodes ricinus*, *Ornithodoros erraticus*	Aquaporins	[188,189,190,193]
Tick Cell Signal Transduction			
	Multiple tick species	Subolesin widely conserved in ticks	[195]
	*Rhipicephalus (Boophilus) australis*	Bm 86 and subolesin	[198]
	*Haemophysalis longicornis*	Subolesin followed by subolesin-PO chimeric polypeptide	[199]
Salivary Gland Proteins			
	*Amblyomma sculptum*	Protease inhibitors of factor Xa, trypsin, thrombin	[204]
	*Rhipicephalus (B.) microplus*	Metalloprotease, rBrRm-MP4	[205]
	*Rhipicephalus appendiculatus*	Attachment cement protein, 64 P	[21,202]
	*Ixodes scapularis*	Salp 15	[207,208]
	*Dermacentor marginatus*	Glutathione S-transferase	[217]
	*Ixodes ricinus*	Serine protease inhibitor	[218]
Tick Microbiome			
	Multiple species	Multiple potential targets for disruption of tick physiological processes with microbiome differences among species and life cycle stages	[219,220,221,222,223,224,225]
	*Ixodes ricinus*	Microbiome Enterobacteriaceae	[34]

## Data Availability

Data sharing not applicable to this article manuscript.
